# Tranexamic acid has no effect on postoperative pain control after arthroscopic rotator cuff repair: A prospective, double-blind, randomized controlled trial

**DOI:** 10.1016/j.asmart.2023.08.003

**Published:** 2023-08-31

**Authors:** Ryosuke Takahashi, Yukihiro Kajita, Yusuke Iwahori, Yohei Harada

**Affiliations:** a Department of Orthopaedic Surgery, Ichinomiya Nishi Hospital, Japan; b Department of Orthopaedic Surgery, Asahi Hospital, Japan; c Department of Orthopaedic Surgery, Hiroshima University, Japan

**Keywords:** Arthroscopic rotator cuff repair, Postoperative pain, Tranexamic acid

## Abstract

**Background:**

The purpose of this study was to compare the efficacies of tranexamic acid (TXA) versus placebo after arthroscopic rotator cuff repair (ARCR).

**Methods:**

This prospective, double-blind, and randomized study was conducted in 70 patients who underwent ARCR from 2021 to 2022 at our hospital. Thirty-four shoulders were randomly assigned to the TXA group, and 36 to the control group; TXA (10 mL) and normal saline (10 mL) were administered locally after surgery and in the control group, respectively. We evaluated visual analog scale pain scores at rest, during activity, and at night and the circumference and diameter of the shoulder joint in both groups preoperatively and at 1, 2, and 3 days, and 1 week after the surgery. We compared and analyzed the results between the groups. Statistical significance was set at a p-value of <0.05.

**Results:**

There was no significant difference in the visual analog scale scores at rest, during activity, and at night between the groups (p > 0.05). The circumference and diameter of the shoulder joint were not also significantly different between both groups (p > 0.05).

**Conclusion:**

Local TXA administration in patients who undergo ARCR does not significantly impact postoperative pain levels and the circumference and diameter of the shoulder joint.

## 1 Introduction

Rotator cuff tear is a common shoulder disorder, with a prevalence ranging from 13% in patients aged >50 years to >50% in those aged >80 years.^1^ Two-thirds of these lesions are asymptomatic, usually owing to their small size, whereas one-third are accompanied by symptoms such as pain, muscle weakness, and limited range of motion.^1^^,^^2^ In symptomatic cases, a rotator cuff tear may lead to restrictions in daily activities. While treatment is mostly conservative, surgical repair of the rotator cuff using arthroscopy is a potential solution in cases refractory to conservative treatment. Among the surgical options available, the incidence of arthroscopic rotator cuff repair (ARCR) has significantly increased recently.^3^^,^^4^ Despite being a minimally invasive procedure, ARCR is usually associated with remarkable postoperative pain in the early postoperative period.^5^^,^^6^ If postoperative pain is controlled well after the operation, patients have a shortened hospital stay and exhibit improved satisfaction and functional recovery.^7^ Therefore, adequate postoperative pain control has become a very important issue for patients who undergo ARCR.

Tranexamic acid (TXA), a commonly used antifibrinolytic, has been used to maintain hemostasis and reduce perioperative blood loss in various orthopedic surgical procedures.^8^, ^9^, ^10^, ^11^, ^12^, ^13^ TXA competitively inhibits plasminogen by reversibly blocking the plasminogen lysine-binding site.^8^^,^^14^, ^15^, ^16^ This mechanism results in stabilization of blood clots through prevention of fibrin degradation.^8^^,^^14^, ^15^, ^16^ This antifibrinolytic agent has become widely popular across multiple subspecialties to decrease blood loss and reduce the need for intraoperative and postoperative transfusions. It has been used in the treatment of several diseases, including hemophilia and von Willebrand disease.^17^^,^^18^

TXA reduces perioperative blood loss and allogeneic blood transfusion rates after arthroplasty surgeries.^14^^,^^19^ Based on the aforementioned evidence, it is reasonable to assume that the hemostatic effect of TXA has the potential to decrease intraoperative, intra-articular, and subacromial bleeding in shoulder arthroscopies, improving intraoperative visual clarity. Although prior studies have reported the efficacy of intravenous administration of TXA in shoulder arthroscopy,^12^^,^^13^ no studies have reported about local administration of TXA after ARCR. This study aimed to evaluate the use of local administration of TXA after ARCR regarding postoperative hemarthrosis and pain. Our hypothesis was that patients treated with TXA would have decreased postoperative hemarthrosis and pain.

## 2 Materials and methods

### 2.1 Study participants

This prospective, double-blind, and randomized controlled trial study involved 70 patients who consecutively underwent ARCR at our hospital between April 2021 and March 2022 and provided informed consent to participate in this institutional review board-approved study. The inclusion criteria were diagnosis of a rotator cuff tear and unresponsiveness to conservative treatment lasting at least 3 months. The tear size of the rotator cuff was evaluated using magnetic resonance imaging (MRI). We measured the longitudinal and transverse dimensions of the tear on preoperative MRI along the oblique coronal and oblique sagittal planes, respectively.^20^ The tear size was categorized as small (<1 cm), medium (1–3 cm), large (3–5 cm), or massive (>5 cm), according to Cofield.^2^ Patients with shoulder osteoarthritis, calcified tendinitis, instability, hemiplegia after stroke, bone metastasis in the shoulder region, history of shoulder fractures or shoulder surgeries, history of coagulopathy and anticoagulation therapy before surgery, and renal or liver disorders were excluded.

Seventy patients were randomly allocated to the two groups: 34 patients were included in the TXA group and 36 in the control group. The TXA group received local periarticular injection of 10 mL TXA (100 mg/mL) into the surgical wound after the surgery. In the control group, the patients were injected with 10 mL normal saline after the surgery, similar to Chiang et al.’s study.^10^ The surgeon, surgical staff members, as well as the patients were blinded to the group to which they were assigned.

**Fig. 1:**
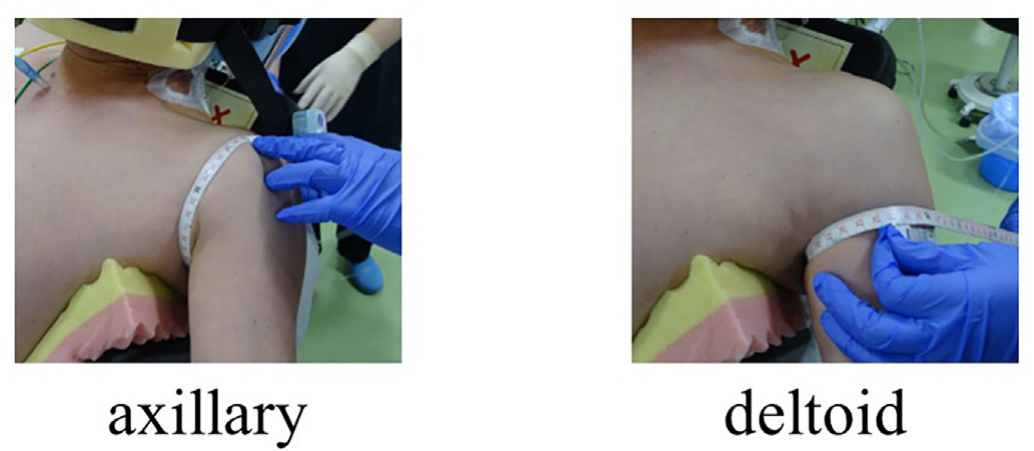
The circumference and diameter of the shoulder joint were measured at the axillary and deltoid sites with the arm abducted approximately 30°.

We evaluated visual analog scale pain scores at rest, during activity, and at night, the circumference and diameter of the shoulder joint in the both groups preoperatively and 1, 2, and 3 days and 1 week after the surgery. The circumference and diameter of the shoulder joint were measured at the axillary and deltoid sites with the arm abducted at approximately 30° (Fig. 1).^12^^,^^13^

### 2.2 Surgical technique

All operations were performed by a single skilled surgeon (Y.K.) uniformly with the patient under general anesthesia and in a beach chair position. Normal saline with epinephrine was used for intra-articular irrigation. The pressure of infusion pump was maintained at 60 mmHg throughout surgery with a pressure-controlled pump. An electrocautery device was used to control bleeding as needed. The arthroscope was inserted via the posterior portal. After visualization, all hypertrophic synovial tissue was cleared. Contracted capsular structures in the rotator interval regions were debrided, and capsulotomy was performed if needed. A standard ARCR was performed using suture anchors; all rotator cuff tears were repaired using the Healix Transtend™ Implant System (Healix BR; Depuy-Synthes Co., Raynham, MA, USA) loaded with 3 high-strength sutures (Versalok Anchor; Depuy-Mitek Inc., Raynham, MA, USA). The number of anchors was decided according to the size of the tear and repair configuration in terms of the suture-bridge repair. Tenotomy or tenodesis was performed in case of a biceps long head lesion. All the patients were administered 1 g acetaminophen intravenously every 6 h for 24 h postoperatively. This repeated dose regimen was chosen based on previously published trials that confirmed its safety and efficacy.^21^

The postoperative protocol was identical in both groups. The shoulders were immobilized for 4 weeks with an abduction brace. Isometric rotator cuff exercises and passive exercises for forward flexion and relaxation of the muscles around the shoulder girdle were initiated the day after surgery. After the immobilization period, active and active-assisted exercises were initiated. After 6 weeks, patients began strengthening exercises of the rotator cuff and scapular stabilizers. Rehabilitation was performed until at least 3 months after surgery with the assistance of a physical therapist. Full return to sports or heavy labor was allowed after 6 months.

### 2.3 Statistical analysis

Paired or unpaired *t*-tests were used to compare the differences in each outcome between the groups after ARCR on the first, second, and third days and at 1 week. Fisher's exact test was used for categorical variables. Statistical significance was set at p < 0.05. All the analyses were conducted using SPSS® v 25.0 (IBM Inc., Armonk, NY, USA).

## 3 Results

The demographic characteristics of the patients are presented in Table 1. There were no significant differences regarding this data between the two groups (p > 0.05).

**Table 1:** Patient's demographics at baseline.

Variables	TXA group	Control group	P-value
Number of shoulders	34	36	
Male/Female	15/19	20/16	0.353
Age (years)	61.9 ± 10.2	61.2 ± 12.6	0.394
Tear size			
below medium-sized	33	34	0.589
above larger-sized	1	2	0.589
The number of rotator cuff tear			
SSP only	27	30	0.763
SSP + ISP	0	1	0.328
SSP + SSC	7	5	0.535
Procedure of biceps long head			
tenotomy	16	14	0.63
tenodesis	1	3	0.615
nothing	17	19	0.816

There was no significant difference in the visual analog scale scores at rest, during activity, and at night between the TXA and control groups (TXA/Control group; 16 ± 16.5, 56.2 ± 23.4, 38.2 ± 30.4/10.8 ± 13.5, 48.4 ± 22.3, 44.4 ± 24.5, p = 0.134, 0.144, 0.243 preoperatively, 27.6 ± 19.4, 45.2 ± 26.5, 65.3 ± 30.3/25.3 ± 20.7, 43.1 ± 31.1, 65.9 ± 34.5, p = 0.335, 0.394, 0.471 for postoperative day 1, 20.2 ± 15.5, 41.6 ± 19.2, 46.9 ± 24.9/20.5 ± 20, 40.5 ± 22.9, 56.1 ± 28.9, p = 0.474, 0.415, 0.082 for postoperative day 2, 13.3 ± 15.2, 40.5 ± 26.1, 38.7 ± 23.1/12.3 ± 13.3, 36.4 ± 22.1, 42.9 ± 30.1, p = 0.385, 0.248, 0.267 for postoperative day 3, 7.8 ± 13.9, 28.8 ± 22.2, 23.1 ± 24.7/7.2 ± 11.1, 30.3 ± 18.5, 22.1 ± 22.1, p = 0.428, 0.377, 0.428 for postoperative day 7) (Table 2). The circumference and diameter of the shoulder joint were not also significantly different between the two groups (TXA/Control group; 397.5 ± 29.5, 310.6 ± 31.7/403.4 ± 45, 308.1 ± 35, p = 0.278, p = 0.386 preoperatively, 431 ± 33.3, 340 ± 31.9/442.6 ± 47, 344.4 ± 38.6, p = 0.145, p = 0.329 for postoperative day 1, 439.3 ± 31.7, 345.5 ± 31.9/453.8 ± 45.6, 356.5 ± 38.3, p = 0.086, p = 0.125 for postoperative day 2, 441.7 ± 29.9, 347.5 ± 30.2/457.8 ± 44.1, 355.4 ± 36.9, p = 0.062, p = 0.198 for postoperative day 3, 416.2 ± 32.5, 333.5 ± 27.4/425.2 ± 36.4, 331.2 ± 28.1, p = 0.174, p = 0.378 for postoperative day 7) (Table 3).

**Table 2:** Results of all outcomes in the TXA group and the Control group (VAS scores).

Variables	TXA group (n = 34)	Control group (n = 36)	P-value
Pre-operative VAS scores (points)			
at rest/during activity/at night	16 ± 16.5/56.2 ± 23.4/38.2 ± 30.4	10.8 ± 13.5/48.4 ± 22.3/44.4 ± 24.5	0.134/0.144/0.243
POD1 VAS scores (points)			
at rest/during activity/at night	27.6 ± 19.4/45.2 ± 26.5/65.3 ± 30.3	25.3 ± 20.7/43.1 ± 31.1/65.9 ± 34.5	0.335/0.394/0.471
POD2 VAS scores (points)			
at rest/during activity/at night	20.2 ± 15.5/41.6 ± 19.2/46.9 ± 19.2	20.5 ± 20/40.5 ± 22.9/56.1 ± 28.9	0.474/0.415/0.082
POD3 VAS scores (points)			
at rest/during activity/at night	13.3 ± 15.2/40.5 ± 26.1/38.7 ± 23.1	12.3 ± 13.3/36.4 ± 22.1/42.9 ± 30.1	0.385/0.248/0.267
POD7 VAS scores (points)			
at rest/during activity/at night	7.8 ± 13.9/28.8 ± 22.2/23.1 ± 24.7	7.3 ± 11.1/30.3 ± 18.5/22.1 ± 22.1	0.428/0.377/0.428

**Table 3:** Results of all outcomes in the TXA group and the Control group (circumference diameter of shoulder joint).

Variables	TXA group (n = 34)	Control group (n = 36)	P-value
Pre-operative circumference diameter of shoulder joint (mm)			
deltoid/axillary	397.5 ± 29.5/310.6 ± 31.7	403.4 ± 45.1/308.1 ± 35	0.278/0.386
POD1 circumference diameter of shoulder joint (mm)			
deltoid/axillary	431 ± 33.3/340.1 ± 31.9	442.6 ± 47/344.4 ± 38.6	0.145/0.329
POD2 circumference diameter of shoulder joint (mm)			
deltoid/axillary	439.3 ± 31.7/345.5 ± 31.9	453.8 ± 45.6/356.5 ± 38.3	0.086/0.125
POD3 circumference diameter of shoulder joint (mm)			
deltoid/axillary	441.7 ± 29.9/347.5 ± 30.2	457.8 ± 44.1/355.4 ± 36.9	0.062/0.198
POD7 circumference diameter of shoulder joint (mm)			
deltoid/axillary	416.2 ± 32.5/333.5 ± 27.4	425.2 ± 36.4/331.2 ± 28.1	0.174/0.378

## 4 Discussion

The most important finding of this study is that patients treated with local TXA administration after ARCR did not improve postoperative hemarthrosis and pain.

Previous studies have mentioned the administration of TXA during many orthopedic surgical procedures to reduce perioperative bleeding and postsurgical hemorrhage.^8^^,^^11^^,^^19^^,^^22^ These studies have shown the safety and efficacy of TXA for controlling perioperative and postoperative bleeding in major orthopedic surgeries. However, it remains unclear whether TXA is advantageous in procedures with relatively preserved soft tissues, such as arthroscopic surgery. Karaaslan et al. reported that TXA reduced the amount of postoperative hemarthrosis and pain in the early postoperative period after arthroscopic anterior cruciate ligament reconstruction.^22^ Felli et al. reported that the administration of TXA could reduce the drainage blood volume and postoperative pain during the first 2 weeks.^11^ Chiang et al. reported that intra-articular injection of TXA reduced pain and postoperative hemarthrosis.^10^ Belk et al. reported that patients undergoing shoulder arthroscopy with TXA can be expected to experience improved outcomes and less hemarthrosis-related complications in the early postoperative period compared with non-TXA patients.^23^ Liu et al. reported the effectiveness of intravenous administration of TXA before ARCR.^12^ They reported that the average visual score in the TXA group (2.5 ± 0.2) was better than that of the control group (2.3 ± 0.3) (p = 0.048). The postoperative subjective pain score was significantly lower in the TXA group (3.0 ± 1.5) than in the control group (4.3 ± 2.0) (p = 0.009). Postoperative analgesic usage was also significantly lower in the TXA group (9.6 ± 9.7 morphine milligram equivalent) than in the control group (14.7 ± 13.4 morphine milligram equivalent) (p = 0.037). Our previous study also reported the effectiveness of intravenous administration of TXA before ARCR.^13^ Visual clarity was found to be significantly better in the TXA group than in the control group. However, the circumference and diameter of the shoulder joint, estimated perioperative blood loss, and operative time were not significantly different between the two groups. Moreover, the visual analog scale scores in the TXA group were not significantly lower than that in the control group.

The route of administration of TXA in orthopedic surgical procedures is a topic of controversy. One meta-analysis comparing the effectiveness and safety of intravenous versus local administration of TXA in patients undergoing total knee arthroplasty showed that both intravenous and local TXA had comparable efficacy in reducing blood loss and blood transfusion rates.^24^ Another meta-analysis on total hip and knee arthroplasty also demonstrated similar results.^25^ Kuo et al. reported that both intravenous and local methods of administration of TXA in shoulder arthroplasty were effective in reducing blood loss and transfusion rates.^26^The potential benefit of local administration is the availability of a higher local concentration of the drug, which may prevent adverse systemic effects.^9^^,^^27^ Another study stated that intra-articular TXA administration in patients undergoing total knee arthroscopy was rapidly absorbed, maintaining a biological half-life of approximately 3 h within the joint fluid compared to intravenous TXA administration.^28^ TXA is increasingly being used in sports medicine surgery, in both knee and shoulder arthroscopy.^10^^,^^12^^,^^13^^,^^23^ However, the present study demonstrated that patients treated with local TXA after ARCR did not report a reduction in postoperative hemarthrosis and pain. In arthroscopic shoulder surgery, TXA may improve visual clarity, while improvements in other bleeding-related outcomes, such as hemarthrosis and total blood loss, may be negligible. This is due to the minimal amount of bleeding that occurs in shoulder arthroscopy as compared with that in open procedures. Moreover, it has raised concerns that a large volume of saline must be pumped into the operative field and locally used TXA is washed out immediately from the wound site in shoulder arthroscopy. This is why there are no significant differences in the visual analog scale and the circumference and diameter of the shoulder joint between the groups. Studies comparing the clinical outcomes of intravenous and local TXA administration in patients undergoing ARCR may be necessary. Moreover, the optimal dosage, route, and timing of TXA application in ARCR surgery remain to be investigated. Further study is also needed on whether the use of TXA affects postoperative pain in shoulder arthroscopy with a much larger patient population.

There are several limitations to our study. First, the sample size was small, and the follow-up period was only 1 week after surgery, which was relatively short in terms of clinical evaluation. It is possible that increased numbers may yield different results. Second, the short-term nature of this study does not provide any information about whether TXA use in ARCR produces better clinical outcomes. Third, this study cannot make any conclusions regarding the optimal dose of TXA. Further head-to-head trials comparing different doses of TXA in shoulder arthroscopy are required to determine the optimal dose.

In conclusion, local administration of TXA in patients who undergo ARCR does not significantly impact postoperative pain levels and the circumference and diameter of the shoulder joint.

## Funding

None.

## Declaration of competing interest

None.
